# Reducing Alcohol Consumption Among Risky Drinkers in the General Population of Sweden Using an Interactive Mobile Health Intervention: Protocol for a Randomized Controlled Trial

**DOI:** 10.2196/13119

**Published:** 2019-04-18

**Authors:** Marcus Bendtsen, Jim McCambridge

**Affiliations:** 1 Department of Medical and Health Sciences, Linköping University Linköping Sweden; 2 Department of Health Sciences University of York York United Kingdom

**Keywords:** telemedicine, alcohol drinking, randomized controlled trial

## Abstract

**Background:**

Harmful use of alcohol continues to be a leading contributor to premature deaths globally. Not only does harmful drinking have consequences for the individuals consuming at increased levels, but it may also result in a range of negative consequences for their family members and friends. Interventions delivered via mobile phones (mobile health [mHealth] interventions) could potentially support risky drinkers seeking help to reduce their alcohol consumption.

**Objective:**

This protocol describes a randomized controlled trial that aims to validly estimate the effect of a novel mHealth intervention targeting risky drinkers in the general population of Sweden. Nested within the trial are 3 substudies that focus on methodological and user satisfaction research questions.

**Methods:**

A 2-arm parallel group randomized controlled trial will be employed to estimate the effect of the novel intervention. Participants will be recruited through Web advertisements and social media. The inclusion criteria are as follows: 18 years or older, ownership of a mobile phone, and being classified as a risky drinker according to Swedish guidelines. Participants allocated to the intervention group will receive a novel mHealth intervention. The intervention consists of weekly screening, personalized feedback on current consumption, functions allowing for planning of future consumption, as well as a series of messages delivered throughout the week. Participants allocated to the control group will receive a short message regarding negative consequences of alcohol consumption and a hyperlink that offers more information. Following 2 and 4 months after randomization, both groups will be asked to complete follow-up questionnaires (2-month interval being primary). Primary outcomes are weekly alcohol consumption and heavy episodic drinking. Participants in the control group will be given access to the novel intervention after completing the 4-month follow-up. The trial includes 3 substudies: We will explore whether the mode of presenting information before participants giving informed consent affects participation rates and recall of trial parameters, investigate if the content of the short message received by the control group affects study outcomes and requests for more information, and explore user satisfaction with the intervention and reactions of the control group.

**Results:**

Participant recruitment is planned to begin in April 2019 and to last for a maximum of 24 months. The first dataset will be available approximately 2 months after the final participant has been recruited, and the final dataset will be available approximately 2 months later. No participants had been recruited at the time of submitting this protocol.

**Conclusions:**

If found effective, the intervention has the potential to reduce negative consequences of alcohol consumption for individuals. The technology has been designed to have potential for extensive reach among those who may benefit.

**Trial Registration:**

ISRCTN Registry ISRCTN48317451; http://www.isrctn.com/ISRCTN48317451 (Archived by WebCite at http://www.webcitation.org/779tKLsu3)

**International Registered Report Identifier (IRRID):**

PRR1-10.2196/13119

## Introduction

### Background

Harmful use of alcohol contributes to approximately 4.5% of deaths globally, as well as having a causal relationship with a range of mental and behavioral disorders [1]. Premature death is not the only negative consequence from harmful alcohol use but it may also lead to injuries, road traffic accidents, violence, and social and economic burdens [2]. Not only does harmful drinking have consequences for the individuals consuming at increased levels but it may also result in a range of negative consequences for their family members and friends.

In Sweden, risky drinking is defined as either drinking more than 9 (female) or 14 (male) standard units of alcohol per week (weekly consumption) or drinking more than 4 (female) or 5 (male) standard units of alcohol on a single occasion at least once a month (heavy episodic drinking). A standard unit is defined as 12 g of alcohol in Sweden. These criteria vary among countries, where for instance the National Institute on Alcohol Abuse and Alcoholism in the United States uses the same thresholds except for a 7-unit threshold per week for women, but at the same time also defines 1 standard unit as 14 g of alcohol. A recent report showed that, in 2016, approximately 31% of the adult Swedish population were risky drinkers according to the Swedish criteria [3].

### Electronic Health and Mobile Health Interventions

Early initiatives to use electronic health interventions to support change of alcohol consumption behavior investigated the use of electronic screening and brief interventions (eSBIs) [4-8]. Commonly, individuals engaging with this type of intervention respond to a series of questions, after which a summary of their drinking habits is presented, and feedback is given with regard to recommended drinking levels, alongside some advice on behavior change.

In a trial including university students in Sweden [7], there were 3.7 percentage points fewer risky drinkers among those who had been invited to complete an eSBI (n=4969) compared with those who had not been invited (n=4972), measured 3 months after initial invitation (odds ratio [OR] 0.91, 95% CI 0.82 to 1.02; *P*=.08). In addition, meta-analyses suggest that there exists a small positive effect of eSBIs on the amount of alcohol consumed weekly in the short term: Cohen *d*=−0.17, 95% CI −0.27 to −0.18 [9]; Cohen *d*=−0.14, 95% CI −0.24 to −0.03 [10]; and weighted mean difference of alcohol in grams=−16.59, 95% CI −23.70 to −9.48 [11]. Although long-term effects have not been measurable, these brief interventions are nevertheless useful for reaching a large number of individuals at a relatively low cost.

Interventions that have attempted to increase effect sizes and make benefits more persistent by requiring participants to revisit a website several times have had problematically low retention [12,13]. However, with the advent of mobile technology, it is now easier to deliver interventions to individuals over time, allowing interventions to become a part of individuals’ everyday life. For instance, it is possible to remind participants of their decisions to reduce their alcohol consumption just before the weekend or ask them to reflect on their consumption on a Sunday evening. Such approaches appear promising from studies of mobile health (mHealth) interventions for behavior change more widely [14-20].

#### Short Message Service–Based Interventions

Interventions that use short message service (SMS) messages to deliver textual content to individuals trying to quit smoking have been widely successful, and the evidence is strong in favor of such interventions [21,22]. However, for alcohol use, the evidence for this type of intervention is less well-developed.

One study invited university students in Sweden to a 2-arm randomized trial comparing a novel SMS-based intervention against treatment as usual (eSBI) [23,24]. The novel intervention consisted of a series of supportive messages sent over 8 weeks. Of the 896 randomized participants, 91.1% (816/896) responded to the 3-month follow-up; however, no significant difference was found between the 2 arms.

Another study investigated the efficacy of a combined Web- and SMS-based intervention to reduce risky drinking among vocational and upper secondary school students in Switzerland (n=1041) [25]. The intervention consisted of a single session eSBI followed by 3 months of SMS messages. Despite losing only 7.01% (73/1041) to follow-up at 6 months after randomization, there were statistically significant differences between those who did and did not respond (education level and estimate of peer’s consumption). Analyses with imputed values did show in favor of the intervention with regard to heavy episodic drinking (OR 0.62, 95% CI 0.44 to 0.87; *P* <.01) but not when complete case analyses were used (OR 0.79, no CI reported; *P*=.24). Therefore, although the evidence may be suggestive of a positive effective, no conclusive evidence was found in favor of the intervention.

Young adults were also randomized in a third study of an SMS-based intervention, drawing its study population (n=765) from emergency department patients at 4 hospitals in Pittsburgh (United States) [26]. One group was given an intervention, which asked them to respond to drinking-related questions and receive feedback through SMS each Thursday and Sunday; another group was asked to respond to drinking-related questions on Sundays only and without feedback; and a control group was not sent any SMS. At 3-month follow-up, 78.0% (597/765) of randomized participants responded, and those who were lost-to-follow-up were more likely to be African American, not currently enrolled in college, and with a baseline higher number of episodes of heavy drinking. Thus, although the trial did identify a significant difference between the participants that received an SMS on Thursdays and Sundays compared with the control who received nothing (drinks per drinking day: incidence rate ratio=0.86, 95% CI 0.79 to 0.94; no *P* value reported), these results should be viewed with some skepticism given the systematic loss to follow-up.

Finally, a pilot study conducted among risky drinkers seeking treatment on the Web suggested that delivering motivational SMS messages daily rather than weekly reduced consumption [27]. Although the study was a pilot, the recruitment of 661 eligible participants over an 8-month period was encouraging.

#### Mobile Phone Apps

The efficacy of alcohol consumption interventions delivered via mobile phone apps has also been investigated in randomized trials. The nature of such apps vary, from simple calculators of estimated blood alcohol concentration to intervention programs that are richer in content.

In Sweden, 2 apps were tested against each other as well as against a control [28]. Participants were university students who screened as risky drinkers (n=1932). Loss to follow-up at 7 weeks was high (from 22.7% [147/649] to 39.1% [250/640] in the different groups), and there were significant signs of systematic attrition, further made problematic by the choice to use per-protocol analyses. The report did not suggest that the apps were effective in reducing alcohol consumption but did, however, suggest that the use of one of the apps might have increased the alcohol consumption of male participants.

Another trial aimed to identify effective components of a mobile phone app [29] and recruited participants (n=672) who were looking for an app (rather than recruiting and then offering an app). A factorial design allowed testing of several variations of the mobile phone app, including both basic and enhanced versions of components. Although the factorial design allowed for important questions to be answered with regard to effective content, a low follow-up rate of 26.6% (179/672) questions any findings from the trial, and the report did not suggest any effect of the intervention on alcohol consumption.

Finally, 1 trial aimed to determine the efficacy of a mobile phone app offering continued support to patients leaving alcohol use disorder treatment [30]. A total of 349 participants were recruited from the Midwestern and Northeastern United States and subsequently randomized to an intervention and control group. The app contained both static content (frequently asked questions, Web links, and daily thoughts) and interactive features (discussion groups, ask an expert, and a weekly brief survey). Follow-up at 4 months identified a significant difference between the control and intervention group with regard to the number of days with heavy episodic drinking (mean difference 1.37; 95% CI 0.46 to 2.27; *P*=.003). No attrition analysis was supplied, but sensitivity analyses only reversed the outcome when missing outcome data (12.3% [43/349]) were set to the maximum possible value.

#### Mobile Health and Alcohol

The development of mHealth interventions targeting harmful alcohol consumption is still in its infancy [31], and there is much that we do not know with regard to increasing effect sizes. A recent meta-analysis determined that the evidence for the effect of mobile phone–based interventions on alcohol consumption reduction was inconclusive [32].

The novel intervention that we are proposing draws from the evidence of eSBIs and leverages technological advancements and the ubiquitousness of mobile phones. Using SMS, we schedule weekly screening of current consumption patterns and offer personal and interactive feedback and advice. In addition, the intervention offers means to plan and set goals for behavior change. Messages are sent via SMS throughout the week to further support a change of alcohol consumption behavior. A more detailed description of the intervention can be found in the section Intervention Content.

### Control Conditions

The estimated effect of an intervention measured in a randomized controlled trial is always to be understood as being relative to the control condition. Attention to the design of control conditions in trials is underdeveloped [33-35]. Being denied immediate access to an intervention, where that has been the motivation for study participation, may have effects on control group participants, which are highly relevant to the interpretation of any apparent intervention effect [36].

It is common to use basic health information (in the form of a leaflet or referring to a website) as a control condition in behavioral intervention trials. Study participants are likely to have previously searched, or in the future search, for alcohol information on the Web. Much information is available of variable quality, and there is a paucity of evaluation studies of the actual effects, if any, of such material [37,38]. Nonetheless, alcohol and health information is commonly used as a control condition in trials, and the design of such control conditions is rarely studied.

### General Data Protection Regulation and Recall of Trial Procedure

With the introduction of the General Data Protection Regulation (GDPR) in the European Union, greater emphasis has been placed on individuals’ rights to their personal data. In layperson’s terms, GDPR says that each individual owns their own personal data, and those who collect it for scientific purposes only borrow it. Individuals have the right to know how the data are going to be used; thus, it is timely to consider issues associated with standard consent practices to develop stronger methods.

Although it may appear easy to obtain consent in internet-based studies, the extent to which typical consent procedures are ethically satisfactory can be questioned. Challenges include conveying that the intervention being evaluated has yet to be shown to be effective, potentially also the concept of placebos or other aspects of the design of control conditions, and allocation to different groups by means of randomization [39,40]. Trial participants’ legal and ethical rights to be offered full disclosure of trial procedures before freely accepting or declining participation is an important aspect of trials involving human subjects; however, poor recall of these trial procedures is troubling [39-42].

### Aims

The overall aim of this study is to evaluate a novel intervention for help-seeking risky drinkers among the general adult population of Sweden in a rigorously designed randomized controlled trial. Nested within the study are 3 substudies. The first substudy aims to investigate participants’ recall of the trial procedure, which they were given information about before confirming their consent. We shall explore two different modes of presenting information regarding the trial procedure. The second substudy will investigate the nature of the control condition. The control group will be given brief health information and have access to further information and then wait for access to the intervention, and we will explore 2 contrasting approaches to the presentation of basic health information. The third substudy will investigate intervention group participants’ experiences of using the novel intervention and control group participants’ reactions to being allocated to the control setting.

The key objectives of the study are to:

1. Validly estimate the effect of the novel intervention on alcohol consumption among risky drinkers in the general population of Sweden in comparison with a health information control condition.
2. Estimate to which degree the total effect is mediated through motivation, importance, and know-how.
3. Explore participants’ recall of trial procedures and if these differ between 2 modes of presenting information.
4. Explore the scope for effects of the 2 contrasting approaches to the presentation of basic health information.
5. Investigate usability and acceptability of the novel intervention in terms of users’ experiences.

## Methods

### Intervention Content

There is no clear picture of which components have the strongest evidence base for inclusion in an alcohol intervention of the kind envisaged. Changing any behavior is a complex process in which many factors interact. Health behavior, from around 30 years ago, has been understood through social cognitive theories of behavior, such as the Health Belief Model and Theory of Planned Behavior. Common to many such theories is the importance of an individuals’ own motivation and self-efficacy, including the skills that the individual possesses and environmental constraints on these intraindividual phenomena [43]. Improving motivation and self-efficacy, as well as teaching new skills and addressing environmental constraints, is understood to improve the likelihood of successful behavior change, including for changing one’s drinking.

The novel intervention that we are proposing aims to target the 4 aforementioned components through the use of several interactive modules contained within a dashboard that individual’s access through their mobile phone. Although the evidence is not yet strong, promising components include those that focus on behavior substitution, problem solving, goal setting, review of behavioral goals, self-monitoring, and normative feedback [44,45]. Thus, modules included in the proposed intervention will revolve around these particular activities. Furthermore, the intervention will include content that aims to increase the understanding of the consequences of alcohol consumption and simulation possibilities that aim to help individuals visualize the outcome of changing their consumption levels.

Each week, participants will receive an SMS that will contain a hyperlink to a dashboard made available on the participant’s mobile phone. The dashboard will allow participants to explore their current consumption, set goals and monitor progress, create plans, learn skills, and learn about the risks involved with alcohol consumption. The dashboard will also work as a simulation device, allowing participants to enter different levels of consumption and interactively seeing health risks change, for example, reducing the number of heavy drinking episodes leads to fewer injuries and reduced risk of premature death. As additional support, participants will receive SMS messages throughout the week that contain motivational and reinforcing information to help them reduce their consumption.

In the following description of the intervention content, we will use behavior change technique (BCT) codes defined in the BCT Taxonomy v1 [46]. This allows us to highlight which techniques are included in the intervention while also creating a link between content and theory.

**Figure 1:**
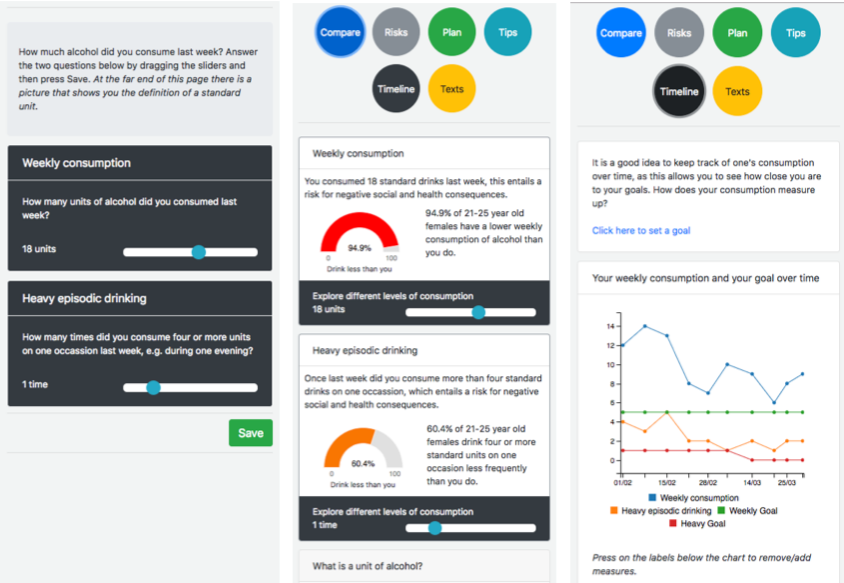
Three screenshots from the interactive dashboard. Left: Each week participants are asked to assess their current consumption. Middle: One of the modules explores normative comparison of consumption levels. Right: Participants can see their consumption over time, allowing them to compare with their own goals.

Weekly SMS messages sent on Sunday evenings will remind participants to access the dashboard and at the same time assess their current consumption. Participants will be asked questions concerning their total weekly consumption and their frequency of heavy drinking over the past week. A screenshot of this is shown to the left in Figure 1. On Wednesdays, Fridays, and Saturdays, participants will receive SMS messages with content aimed to increase motivation and skills. The message set is a refinement of a previously developed set that was created through formative development, and a BCT analysis of these messages has been conducted and reported previously [47].

Apart from the SMS messages and weekly assessments, the dashboard will have 6 modules, which can be accessed by participants in any order and any number of times:

- Normative comparison of the participants’ current consumption compared with others of the same age group and gender (based on data from Sweden). A screenshot of this module can be found in the middle of Figure 1. At the bottom of the module, participants can change consumption levels, which changes the normative feedback interactively, effectively allowing participants to explore how different levels of consumption lead to different outcomes. This module will leverage BCT6.2 and BCT9.3, which concerns social comparison (normative feedback) and comparing future outcomes.
- One module will give information about general risks and risk of disease connected to different levels of alcohol consumption. This module will also allow participants to simulate different levels of consumption and interactively see how risks change. This module is connected to BCT9.3 (comparing future outcomes) as well as BCT5.1 and BCT5.3, which addresses information about both social and health consequences of excessive alcohol consumption.
- One module will allow participants to create a plan that they can use when subjected to a behavioral trigger (eg, going to the pub). This module will ask participants to write an SMS message to themselves and pick a time and date for when they want to receive this message in the coming week (up to 3 times). This module leverages BCT1.2 (problem solving).
- General tips to strengthen participants’ know-how on how to reduce their consumption will be given in 1 module. The tips will leverage BCT7.2, which suggests that participants should create prompts or cues, for example, putting a ribbon around their wrist as a reminder that they have committed to reduce their drinking, as well as BCT8.1 and BCT8.2, which suggest that participants practice a new behavior and substitute their current behavior with a different one (eg, replacing at least 2 alcoholic beverages with nonalcoholic beverages each week). The tips will also concern identification of relapse triggers and barriers, avoiding social cues for drinking, and environmental restructuring (eg, avoid keeping alcohol at home).
- One module will show the participants’ consumption over time. A screenshot of this module can be found to the right in Figure 1. The data come from the weekly assessments. This module leverages BCT2.2 and BCT2.3, which concerns recording and feedback of performance over time. Optionally, participants can decide to set a goal for their consumption, which would then show up graphically in the chart. This will allow participants to set and review their own goals while also visualizing the discrepancy between their current consumption and their goals (BCT1.1, BCT1.5, and BCT1.6).
- The final module will allow participants to sign up for additional SMS messages sent to their mobile phones throughout the week (ie, until the next assessment). This will add SMS messages on top of the messages already received on Wednesdays, Fridays, and Saturdays.

At any time, participants can send an SMS message with the word STOP to the phone number from which they receive messages. At this point, there will be no more SMS messages sent to the participant, including weekly assessments and links to the dashboard. Participants will, however, receive 1 more message where it says that we have acknowledged that they no longer wish to receive the intervention, unless they respond with START in an SMS, and that we will contact them solely with follow-up questionnaires, as previously agreed. The 4-month period of study for this trial is for research purposes only as, in principle, there is no finite end point to the intervention, and in a real-world setting, participants could engage with the intervention as long as they prefer. Therefore, we do not strictly interpret a STOP message from a participant as noncompliance but rather that the individual has decided that they no longer need the support. In an exploratory analysis, we will investigate if there is a relationship between alcohol outcomes and those who decide to stop the intervention before the 4-month mark.

### Design

To study the effect of the intervention, a 2-arm parallel group randomized controlled trial will be employed. Nested within this trial will be the 3 substudies.

#### Recruitment and Eligibility

**Figure 2:**
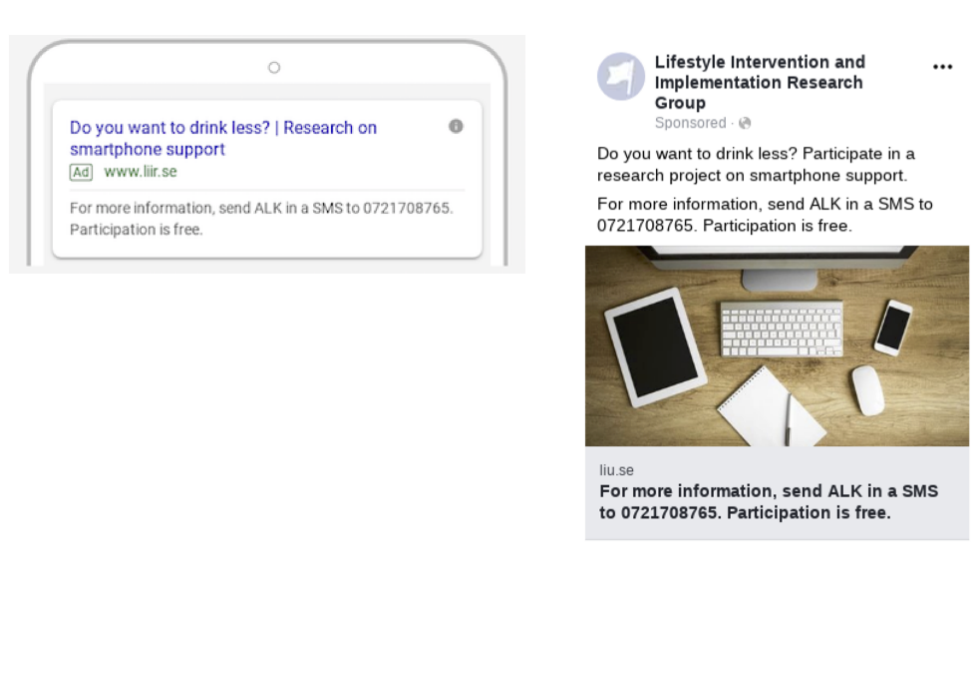
Advertisements shown in search and on Facebook.

Using Web search engine advertisements (Google, Yahoo, and Bing) and social media (Facebook), we will target individuals in the general population of Sweden seeking help to reduce their alcohol consumption (advertisements are shown in Figure 2). Participants will initially be recruited over a 6-month period. Additional 6-month periods will be added if not enough participants have been recruited according to the initial power calculation. If 20.03% (426/2126) or less of the required population has been recruited at 12 months, then recruitment will stop at this time and data will be analyzed to inform future trial design. Recruitment will stop after 24 months if not before.

Individuals interested in participating in the study will be asked to send an SMS message with a specific code to a dedicated phone number. Within 10 min, participants will receive an SMS with a hyperlink that takes them to a Web page asking for informed consent. Individuals will be randomized to 1 of 2 different means of presenting the trial procedure when asking for informed consent (Consent-1 and Consent-2). Please see Multimedia Appendix 1 for more details.

All individuals giving informed consent will be asked to complete a baseline questionnaire, which will also assess eligibility for the trial. The inclusion criteria will include the following: aged 18 years or older, ownership of a mobile phone, and being classified as a risky drinker according to Swedish guidelines.

Note that there is no upper limit to alcohol consumption as an exclusion criterion, meaning the study population is anticipated to comprise both harmful (ie, individuals who have experienced harm from any level of consumption [48]) and hazardous drinkers (ie, individuals with a consumption pattern that suggests increased risk to health [48]). No additional support will be offered to individuals who have a very high-consuming behavior.

#### Intervention and Control Conditions

Eligible individuals will be randomized to either an intervention group or a control group (intervention and control). The intervention group will receive the novel intervention for 4 months and will also be recommended to read about alcohol, health, and society on the same website as the control group (see Multimedia Appendix 2). Participants allocated to the control group will be advised that they will go through an initial phase of 4 months during which they are to receive information to increase their motivation and reduce their consumption, after which they will be given access to the new support tool. Thus, individuals allocated to the control setting will be given access to the novel intervention after completion of the final follow-up (4 months after randomization); no further data will be collected from individuals in the control setting.

The control group will be randomized further into 2 groups (Info-1 and Info-2). Both Info-1 and Info-2 will receive a single SMS message with basic health information regarding short- and long-term effects of alcohol consumption. In this substudy, we incorporate a contrast between 2 very brief types of information; one which emphasizes possible complexities associated with the short- and long-term effects of alcohol (such as is widely available from alcohol industry sources [49], Info-1) and another which provides a clear and straightforward public health messaging style (while being appropriately evidence informed, Info-2). Each is delivered in a single SMS and includes the same link to a Swedish website with information about alcohol and society (IQ). As individuals who have enrolled are looking for help to change their alcohol consumption, we anticipate that most participants will be motivated to click on the link. It is plausible that either type of message could encourage trial participants more than the other to click on the link. This exploratory substudy concerned with the direct behavioral effects on accessing health information will assist further consideration of the design of control conditions and be relevant to alcohol health promotion more broadly. As there is some health information provided, including to participants who do not click on the link, we refer to the control condition as alcohol information. Please see Multimedia Appendix 2 for more details.

#### Mediation

To further understand the potential effects of the proposed intervention, we will measure psychosocial factors believed to be important for behavior change. We will use these measures to estimate how the effects of the intervention are mediated through these factors. Specifically, we will measure perceived confidence, importance, and know-how.

Confidence is closely linked to self-efficacy, a cornerstone of modern theoretical models of behavior change, prominently in social cognitive theory [50]. Importance is an aspect of both motivation and intention, 2 key factors in modern theory, for example, protection motivation theory [51], social cognitive theory [50], and theory of planned behavior [52] (and has been retrospectively added to the health belief model [53]). Know-how, alternatively expertise or skills, connects with several factors in theoretical models, for example, to behavioral control in the theory of planned behavior, and has been proposed as a specific necessary factor for behavior change [43,54]. Measuring if, and to what degree, these factors mediate the effect of the proposed intervention will help us better understand which factors are affected by the intervention and thus, why the intervention (potentially) works.

#### Randomization

In all cases, randomization will be fully computerized, will not employ any strata or blocks, and will not be possible to subvert as this and all subsequent study processes are fully automated. The main study will be single-blind as participants will be aware that 2 settings exist, and they will know which one of them they have been allocated to (intervention or control). The substudies involving Consent-1, Consent-2, Info-1, and Info-2 will be double-blind as participants will not be aware of the existence of the substudy or that they have been randomized. It is not possible to blind the individual responsible for data analysis, as this individual will also be responsible for data collection and involved in the monitoring of the technical platform.

#### Follow-Up

At 1 month after randomization, an SMS message will be sent to both intervention and control with a hyperlink to a questionnaire exploring mediators. At 2 and 4 months after randomization, an SMS message will be sent to both intervention and control with a hyperlink to a questionnaire exploring alcohol consumption. At 2 months after randomization, the questionnaire exploring recall of trial procedures will be added for both groups. At 4 months after randomization, the intervention group will receive questions regarding the usability and their experiences of the intervention, and the control group will receive questions regarding their experiences of being allocated to the control condition.

**Figure 3:**
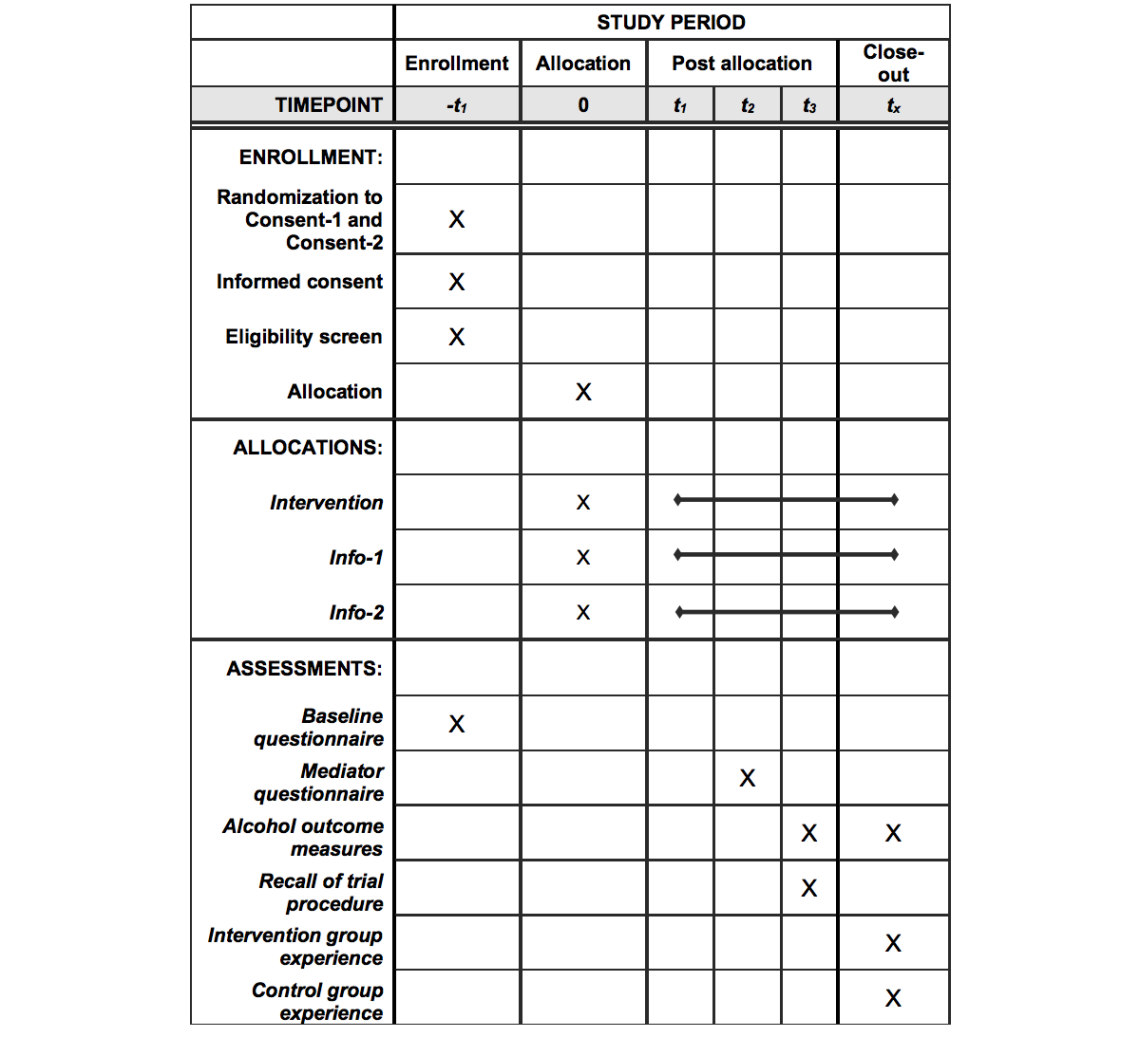
Standard Protocol Items: Recommendations for Interventional Trials figure.

**Figure 4:**
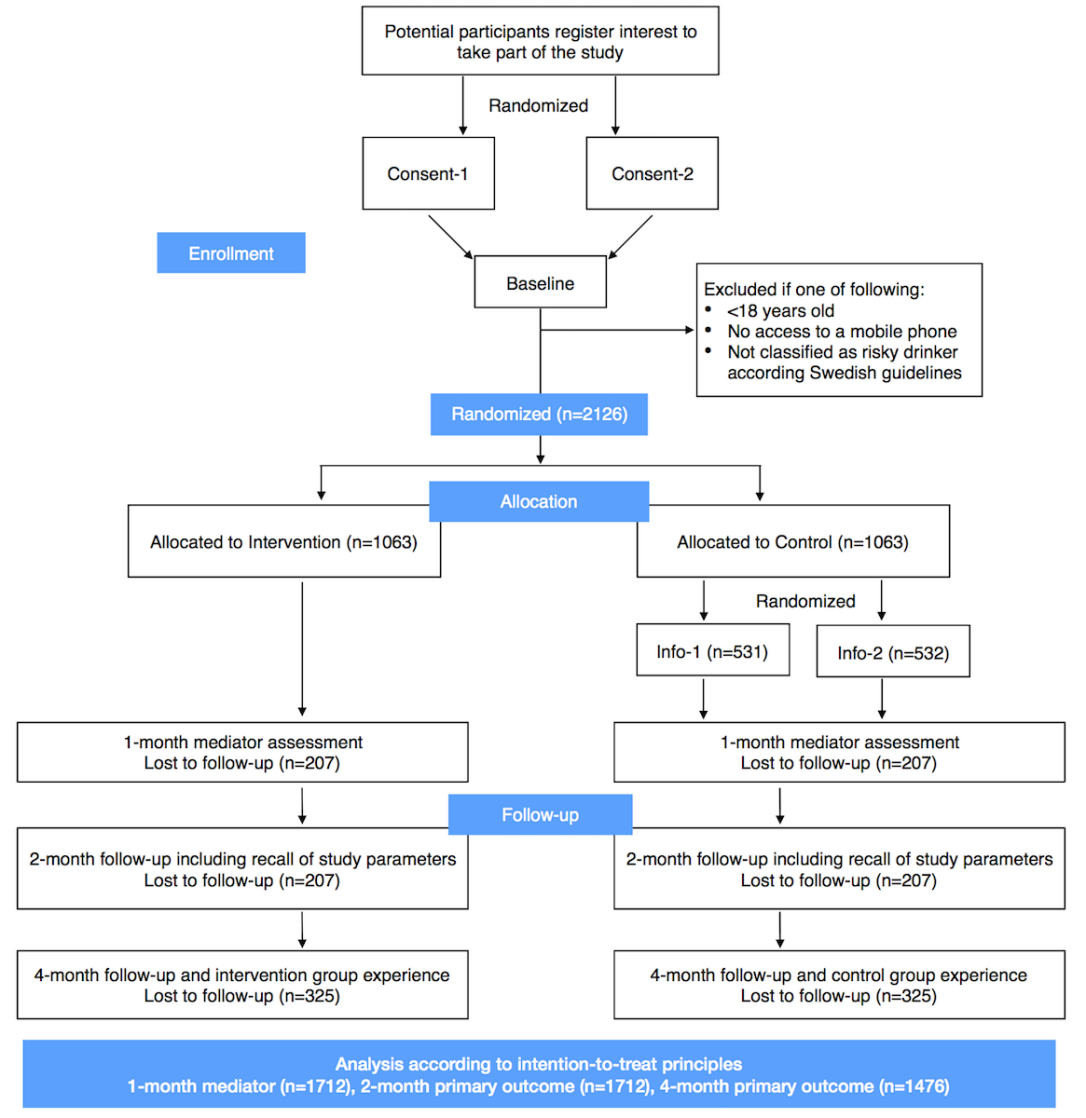
Consolidated Standards of Reporting Trials flow diagram (see power calculation section for details of n).

Figures 3 and 4 offer an overview of the design in the form of a Standard Protocol Items: Recommendations for Interventional Trials figure and Consolidated Standards of Reporting Trials flowchart.

### Hypotheses

There are 5 main and 2 exploratory hypotheses mentioned in Textbox 1.

Apart from the hypotheses tested here, we will also do both a quantitative and qualitative analysis of users’ experiences of engaging with the intervention and being allocated to the control group.

### Measures

All questions asked at baseline and subsequent follow-ups can be found in Multimedia Appendix 3.

For hypotheses 1, 6, and 7:

- Primary outcome measures: Total weekly alcohol consumption and frequency of heavy episodic drinking.
- Secondary outcome measures: Classification as risky drinker according to Swedish guidelines.

For hypothesis 2:

- Mediation measures: Confidence in one’s ability to reduce consumption, importance of reducing, and knowledge of how to reduce consumption.

For hypotheses 3 and 4:

- Primary outcome measures: Enrollment rates from each study condition
- Secondary outcome measures: Recall of trial procedures measured through a series of questions.

For hypothesis 5:

- Primary outcome measures: Rates of additional information being requested by pressing on the supplied hyperlink.

Total weekly alcohol consumption will be assessed by asking participants the number of standard units of alcohol they consumed last week, that is, a short-term recall method [55]. Using a summary measure, rather than asking day-by-day, will allow for the same question to be asked, regardless if responses are collected via Web questionnaires, SMS, or by phone interviews (see Follow-up Attempts). In addition, 1 study suggests that when considering short-time spans, summary measures do not imply any noteworthy bias compared with asking for consumption day-by-day [56]. The frequency of heavy episodic drinking will be assessed by asking participants how many times they have consumed more than 4 (females) or 5 (males) standard units of alcohol on 1 occasion the past month, again asking for a summary measure over a concrete time period. An individual is classified as a risky drinker if either total weekly consumption or heavy episodic drinking exceeds recommendations.

Confidence, importance, and know-how will be measured at baseline and at 1-month follow-up by asking “How confident are you that you will be able to reduce your alcohol consumption?,” “How important do you think it is to reduce your alcohol consumption?,” and “How well do you know how to reduce your alcohol consumption?,” all 3 with response options on a 10-point scale (see Multimedia Appendix 3). The wording of the questions at 2- and 4-month follow-up will change to include those who have reduced their consumption: “How confident are you that you will be able to reduce or keep a lower level of alcohol consumption?,” “How important do you think it is to reduce or keep a lower level of alcohol consumption?,” and “How well do you know how to reduce or keep a lower level of alcohol consumption?.”

**Hypotheses.**

**Main Hypotheses**

1. Alcohol consumption will differ between intervention and control groups, with the intervention group drinking less than control at 2 and 4 months after randomization. The 2-month interval will be primary.

2. Confidence, importance, and know-how at 1-month follow-up will mediate the effects of the intervention on drinking outcomes at 2-month follow-up. The same measures at 2-month follow-up will mediate the effect on drinking outcomes at 4-month follow-up.

3. Enrollment rates will differ between groups Consent-1 and Consent-2.

4. Accurate recall of study parameters will differ between groups Consent-1 and Consent-2.

5. Rates of accessing further information will differ between groups Info-1 and Info-2.

**Exploratory Hypotheses**

6. Alcohol consumption will differ between groups Consent-1 and Consent-2 at 2 and 4 months after randomization. The 2-month interval will be primary.

7. Alcohol consumption will differ between groups Info-1 and Info-2 at 2 and 4 months after randomization. The 2-month interval will be primary.

With regard to recall of trial procedures, there are 3 parameters that are of interest. First, participants will be asked if they recall receiving information about allocation to 2 different groups. As information regarding group allocation was presented identically for Consent-1 and Consent-2, this question will both work as a check that randomization succeeded and as a general baseline for recall regardless of group allocation. Second, participants will be asked if they recall how personal data will be handled in the study and their rights to these data. Finally, participants will be asked if they remember how the data would be analyzed and results communicated (see Multimedia Appendix 3).

We wish to measure if participants access further health-related information after reading a short SMS with basic information about short-term and long-term consequences of alcohol consumption. A proximal outcome of this is to measure whether or not participants in Info-1 and Info-2 request more information by following the included hyperlink. In addition, a short questionnaire at 4-month follow-up will investigate the control group participants, which will be used to investigate further differences between Info-1 and Info-2 (see Multimedia Appendix 3).

The acceptability and usability of the intervention will be measured through a questionnaire and the system usability scale [57] at 4-month follow-up (see Multimedia Appendix 3). The system usability scale comprises 10 Likert items exploring users’ perception on usability of a product or service.

### Follow-Up Attempts

There are 3 follow-up stages: 1, 2, and 4 months after randomization. All follow-ups will be initiated by sending SMS messages to participants with hyperlinks to questionnaires. In all cases, the following attempts will be made to collect data from nonresponders:

1. A total of 2 reminders will be sent 2 days apart to those who have not responded.
2. If no response is given to (1), then we will send questions directly in an SMS message, asking participants to respond directly with an SMS (no hyperlink).
  - At 1 month, we will ask all 3 mediator questions.
  - At 2 and 4 months, we will only ask for primary alcohol outcome measures.
3. If there is no response given to (2), we will attempt to call participants to collect responses to the same questions as in (2). A maximum of 5 attempts will be made.

### Statistical Analysis Methods

All analyses will be done under the intention-to-treat principle, where all randomized individuals will be included. Missing outcome data will initially be handled by a complete-case analysis, which assumes that data are missing at random (MAR). If data are systematically missing, then it may be the case that early responders differ from late responders and, in extension, that late responders are more similar to nonresponders. We will, therefore, explore the plausibility of the MAR assumption by regressing the primary outcomes on the number of follow-up attempts needed before a response was recorded. To further explore the MAR assumption, attrition will be investigated among study groups by comparing baseline characteristics between those who did and did not respond at follow-up. A sensitivity analysis that includes imputed values for missing outcome data (using multiple imputation by chained equations [58]) will also be performed. Data will be graphically examined for outliers or data input errors, and sensitivity analyses will be performed excluding any erroneous data points.

For all models, coefficients of interest will be assessed for statistical significance using a null hypothesis testing approach, where tests will be 2-tailed at the .05 significance level. Alongside the null hypothesis tests, posterior distributions using a Bayesian approach will be calculated for each coefficient. Both significance tests and posterior distributions will create a basis for scientific inference [59].

#### Baseline

Baseline characteristics will be compared among the different groups using chi-square tests or Fisher exact tests for comparison of proportions and Mann-Whitney *U* tests for comparison of means.

#### Hypotheses 1, 6, and 7

Total weekly alcohol consumption and frequency of heavy episodic drinking are likely to be skewed and overdispersed, and we will, therefore, analyze both using negative binomial regression. Classification as a risky drinker will be analyzed using logistic regression. Both unadjusted and adjusted regression models will be created. Adjusted models will include baseline values of the respective outcome measures and responses to the baseline questions regarding sex, civil status, age, motivation, importance, and know-how. As alcohol consumption differs significantly among age groups, and possibly also the effect of the intervention, multilevel models will also be created in which slopes are allowed to vary among age groups. Effect modification tests will be performed in all models to assess if any of the baseline characteristics moderate the effect of the intervention. The adjusted models will be the primary models.

#### Hypothesis 2

Mediators will be explored using a causal inference framework [60], where Monte Carlo methods are relied upon for inference. This allows for any type of model (linear and nonlinear) to be used to represent the relationships between the group allocation, mediating variable, and outcome. A total of 4 models will be created for each outcome measure, 3 that investigate the mediating factors on their own and a fourth that incorporates all mediators at once. If any baseline characteristics were found to moderate the effect in the primary analysis, then additional mediator models will be created to include these as moderators.

#### Hypotheses 3 and 4

Whether or not individuals decided to give informed consent will be regressed against group allocation (Consent-1 and Consent-2) using logistic regression. Adjusted regression models will be explored that include baseline characteristics (including only participants who gave informed consent). Differences among the 2 groups on responses to the recall questionnaire will be investigated using chi-square tests or Fisher exact tests for comparison of proportions.

#### Hypothesis 5

Whether or not individuals requested more information by pressing the hyperlinks will be regressed against both group allocation (Info-1 and Info-2) and fixed responses to the control group experience questionnaire using logistic regression. Adjusted models will be explored that include baseline characteristics. Differences among the 2 groups on responses to the control group experience questionnaire will be investigated using chi-square tests or Fisher exact tests for comparison of proportions.

#### Acceptability and Usability of Intervention

Responses to the Likert items of the system usability scale are processed so that the total score ranges from 0 to 100. Empirical and validation studies suggest that systems scoring above 68 should be considered to have an above average usability [61]. We will calculate mean total scores and compare against this general guideline of 68.

To further investigate how different individuals perceive the usability of the intervention, linear regression models will be used to regress system usability scores against baseline characteristics and alcohol- and mediator-related outcome measures, as well as usage statistics of the intervention (collected as participants engage with the intervention).

#### Exploratory Analysis

The weekly assessments of the intervention group are primarily an aid for the individual to his or her behavior change. The data cannot be used in the primary analysis as they will quite certainly contain a lot of missing data (we do not expect participants to zealously report each week) and there are no data from controls. However, the data collected may nevertheless be useful to identify trends in potential reduction of alcohol consumption over time, thus, exploratory time series models will be created to see if there are patterns that are informative about intervention effects (such as reduction plateaus). Similarly, we will regress alcohol consumption outcomes on usage statistics in the intervention group, including frequency of use of different modules and whether or not the participant decided to stop the intervention before the end of the trial, possibly identifying a dose-effect relationship.

As part of the primary investigator’s precision health initiative, we aim to include predictive modelling of the intervention. Traditionally, trials contrast the mean difference between 2 groups; however, they do not address individual variability. Intuitively, we know that some individuals will respond well to an intervention, whereas others might not, and some might further be harmed by it. We wish to predict how individuals will respond to an intervention using only individuals’ baseline characteristics. We do this by measuring characteristics at baseline related to the behavior change theory, in this particular case self-efficacy, importance, and know-how, as well as alcohol consumption levels and conventional baseline characteristics (age and gender). These characteristics are then used to learn statistical models that predict individual outcomes.

Predictive analysis requires a radically different approach of assessing a model’s performance, as explaining and predicting are 2 different tasks [62]. We utilize a Bayesian approach using shrinkage priors [63,64], which allows us to include all characteristics measured at baseline and then learn which ones should be included in the predictive model from the data. The result is a model that can tell individuals how likely it is that the intervention has a positive effect on them specifically, rather than always quoting the group mean difference.

### Power Calculation

We conducted a Monte Carlo simulation study to determine the necessary number of individuals to randomize (N). We deliberately focused on the total weekly consumption outcome, as the analysis of this outcome in general requires more individuals than does the analysis of heavy episodic drinking (because of higher variance). We will begin by describing the initial distributional assumptions that we made and then the result of the simulations. We aimed to achieve 0.8 power at the .05 significance threshold.

- The effect (γ) of the intervention, that is, the percentage reduction in mean total weekly alcohol consumption in the intervention group, is assumed to follow a beta distribution with mean 0.15 and SD 0.025.
- The mean number of standard units consumed in the control group (μ_C_) at follow-up is assumed to follow a normal distribution with mean 10 and SD of 0.5. The population under analysis are risky drinkers, thus, a mean consumption of 10 standard units is appropriate, and the analysis is quite robust to moderate changes in this assumption.
- The mean number of standard units consumed in the intervention group (μ_I_) at follow-up is assumed to follow a normal distribution with mean (1-γ)_C_ and SD of 0.5.
- The distribution over the number of standard units consumed at follow-up for both the intervention and control group is assumed to follow a negative binomial distribution. The means of these distributions are given by μ_C_ and μ_I,_ respectively, and have a dispersion parameter θ sampled from a normal distribution centered at 1 with an SD of 0.05.
- The number of individuals allocated to the intervention group follows a binomial distribution, with a 0.5 probability of success over N trials.

The Monte Carlo simulation explored different values of N (number of individuals randomized). For each N explored, the following simulations were done:

1. Draw 1 sample each for γ, μ_C_, μ_I,_ and θ
  - Allocate a random number of individuals to the intervention
  - Give each individual a number of standard units consumed following the negative binomial distribution appropriate for their respective groups
  - Analyze the data using negative binomial regression
  - Note if the 0.05 threshold has been broken for the group allocation coefficient in the regression model
  - Repeat 100 times from (a) and then calculate power as the percentage of times that the threshold was broken
2. Repeat 1000 times from (1) and calculate the average power from (e)

We found that for N=1800; we have an expected average power of 0.79.

In previous studies similar to this [24,65], we have been able to achieve more than 90% response rate at follow-up using the scheme described in the section Follow-up Attempts. Assuming that the response rate is lowest at 4 months and that it follows a beta distribution with mean 85% and an SD of 5% (giving a 95% credible interval between 75% and 94%), then we will expect to lose 326 individuals to follow-up (interquartile range: 231,398). This implies that we need to randomize approximately 2126 individuals (interquartile range: 2031, 2198).

## Results

Participant recruitment is planned to begin in April 2019 and last for a maximum of 24 months. The first dataset will be available approximately 2 months after the final participant has been recruited, and the final dataset will be available approximately 2 months later. No participants had been recruited at the time of submitting this protocol.

## Discussion

### Principal Findings

In Sweden, the student health care centers routinely administer eSBIs (via email) to the university students that they serve [7,8], and the general public have access to eSBIs via websites (eg, livsstilsanalys.alexit.se). However, beyond this single session intervention, there are no evidence-based digital interventions available for those who need continued support, despite the ubiquitousness of mobile phones in Sweden. The advice for individuals who need support beyond the eSBI is generally to search for advice on health websites or to seek help at a primary health care center.

This study is an evaluation of whether or not access to the novel intervention has any effect on alcohol consumption outcomes compared with providing information including referring individuals on to an alcohol and health website. If found effective, this type of intervention has the potential to reduce the burden from negative consequences of excessive alcohol consumption for individuals who need support beyond a single session eSBI and has been designed to have potential for extensive reach among those who may benefit. It has also been designed so that individuals can choose how long they use it for, meaning that intervention exposure can potentially extend for many years.

Previous trials of mHealth interventions targeting alcohol consumption have been aimed toward specific subgroups of a population, quite often young adults or individuals in alcohol use disorder treatment programs. Many of the studies have had issues with trial design or execution, and therefore, the collective evidence for mHealth interventions’ effect on alcohol consumption is not well-developed. The novelty of targeting the adult population of Sweden by means of a broader recruitment than in previous trials, in the ways proposed, allows for both primary prevention: helping individuals prevent negative consequences, and secondary prevention: helping individuals to reduce negative consequences already experienced. By using a scheme that we have previously found successful in achieving high follow-up rates, we aim to avoid the attrition issues that have manifested in previous studies.

### Substudies

Apart from the main outcome, this trial will also investigate methodological and ethical issues in randomized controlled trials. Although informing participants about trial procedures before asking for informed consent has been required for some time, the introduction of GDPR has increased the focus on data privacy and handling of personal information. Participants should be made aware of their rights, and the findings from the substudy regarding recall of trial procedures will give insights into how well participants read, and later recall, the information given before informed consent. If individuals cannot recall that randomization was to occur, the ethical implications deserve fuller consideration.

Similarly, we will explore the nature of the alcohol information control condition and the extent to which introductory text has implications for accessing further information via a link. This study is informative about the inter-related issues of the possible effects of contrasting types of information and the nature of control conditions, which commonly employ informational content.

### Ethical Concerns

The conduct of the substudies raise ethical issues, as they do not themselves involve informed consent. The possible importance of each substudy provides 1 possible justification for not seeking consent, as seeking consent would interfere with the substudy itself. The consent study also involves data collection from those who choose not to participate in the trial. A key consideration in such a situation is the possibility of harm to participants [66]. In this instance, not obtaining informed consent is regarded as being unlikely to produce harm in each substudy.

A further ethical risk lies in the nature of the control condition and its appropriateness for those who have been targeted for recruitment because they wish to drink less. Ethical considerations led us to construct the control condition, and the information contained within each arm, to resemble content that is available on the Web that study participants may encounter. The control condition is thus similar to usual care. Note, however, that this is not a population that is defined by the existence of problems or has been identified as seeking help to reduce problems, beyond responding to Web advertisements. For those participants who are drinking harmfully or seeking further help, the control condition refers on to the national Swedish Web resource.

### Limitations

The trial is designed to recruit enough participants to power the main alcohol-related outcomes; thus, the included substudies are not powered to detect significant differences. Rather, the substudies included herein are supposed to be seen as preliminary and exploratory work laying a basis for future trials of these phenomena.

The power calculation considers a range of follow-up rates (beta distribution with a mean of 85% and an SD of 5%), and the number of individuals (n=2126) found to be necessary to randomize may have to increase toward the upper quartile (n=2198) if follow-up rates are found to be lower than the mean of 85%. Our previous trials [24,65] have been able to collect data from more than 90% of participants using the same follow-up scheme used in this trial; thus, despite not using incentives to sustain high levels of follow-up rates, we believe our expectation of 85% follow-up rate to be warranted.

## Appendix

Multimedia Appendix 1 Information regarding trial procedures.

Multimedia Appendix 2 Group information.

Multimedia Appendix 3 Questionnaires.

## Abbreviations

BCT: behavior change technique
eHealth: electronic health
eSBI: electronic screening and brief intervention
GDPR: General Data Protection Regulation
MAR: missing at random
mHealth: mobile health
OR: odds ratio
SMS: short message service
