# Supplementary material for: Reducing Alcohol Consumption Among Risky Drinkers in the General Population of Sweden Using an Interactive Mobile Health Intervention: Protocol for a Randomized Controlled Trial
Source: JMIR Res Protoc. 2019 Apr 18;8(4):e13119. doi: 10.2196/13119 (PMC6495288; doi:10.2196/13119)
Supplement: Multimedia Appendix 3 [file resprot_v8i4e13119_app3.pdf]

APPENDIX C – QUESTIONNAIRES

BASELINE QUESTIONNAIRE

1. Sex:
   a. Female
   b. Male
2. Civil status:
   a. Living alone without kids at home
   b. Living alone with kids at home
   c. Living with somebody without kids
   d. Living with somebody with kids
   e. Have a partner but not living together
3. Age (numerical measure)
4. How often, during the past month, have you consumed four/five (female/male) or more standard units on one occasion? (numerical measure)
5. How many standard units of alcohol did you consume last week? (numerical measure)
6. How confident are you that you will be able to reduce your alcohol consumption? (10-point scale ranging from 1 = "Not at all" to 10 = "Very confident")
7. How important is it for you to reduce your alcohol consumption? (10-point scale ranging from 1 = "Not important" to 10 = "Very important")
8. How well do you know how to reduce your alcohol consumption? (10-point scale ranging from 1 = "Not well at all" to 10 = "Very well")

Note: Participants are reminded of the definition of a standard unit by graphical means.

MEDIATION QUESTIONNAIRE (ONE-MONTH FOLLOW-UP)

1. How confident are you that you will be able to reduce your alcohol consumption? (10-point scale ranging from 1 = "Not at all" to 10 = "Very confident")
2. How important is it for you to reduce your alcohol consumption? (10-point scale ranging from 1 = "Not important" to 10 = "Very important")
3. How well do you know how to reduce your alcohol consumption? (10-point scale ranging from 1 = "Not well at all" to 10 = "Very well")

FOLLOW-UP QUESTIONNAIRE (TWO- AND FOUR-MONTH FOLLOW-UP)

1. How often, during the past month, have you consumed four/five (female/male) or more standard units on one occasion? (numerical measure)
2. How many standard units of alcohol did you consume last week? (numerical measure)
3. How confident are you that you will be able to reduce or keep a lower level of alcohol consumption? (10-point scale ranging from 1 = “Not at all” to 10 = “Very confident”)
4. How important is it for you to reduce or keep a lower level of alcohol consumption? (10-point scale ranging from 1 = “Not important” to 10 = “Very important”)
5. How well do you know how to reduce your alcohol consumption? (10-point scale ranging from 1 = “Not well at all” to 10 = “Very well”)

Note: Participants are reminded of the definition of a standard unit by graphical means.

RECALL OF TRIAL PROCEDURE (TWO-MONTH FOLLOW-UP)

Before you accepted to join this trial you were given information about the trial procedure. We would like to ask you a few questions about this information.

1. Which one of these statements most accurately describes your recall of group allocation:

- I recall reading information about two groups, but no details.
- I recall reading information about two groups, and that each group was going to be given access to either information or immediate access to a new mobile phone support tool.
- I do not recall reading about allocation to two groups.

2. (If second option is picked to question 1): Which one of the groups were you allocated to?

- I was given immediate access to information.
- I was given immediate access to a new mobile phone support tool.
- I do not know.

3. With respect to how personal data would be handled during the trial, which of these do you recall reading about? You can select multiple options, and if you cannot recall any then select “I do not recall reading about personal data”:

I recall reading about:

- How data would be stored in connection to my phone number
- How my phone number would be encrypted when stored
- My rights to the data according to GDPR
- Who to contact in case I have a complaint regarding data handling
- How phone numbers were going to be treated once the project is complete

- That my data cannot be traced to my phone number after the project is complete
- I do not recall reading about personal data

4. Which one of these statements most accurately describes your recall of how data collected from this trial would be analysed and the results be made available?

- I recall reading about data analysis and communication of results, but no details.
- I recall reading about data analysis and communication of results, and some details of the analysis part.
- I recall reading about data analysis and communication of results, and some details of the communication part.
- I recall reading about data analysis and communication of results, and some details of both parts.
- I do not recall reading about data analysis nor communication of results.

Note: Each question will also have a free-text area in which participants can leave further comments and impressions.

INTERVENTION GROUP EXPERIENCE (FOUR-MONTH FOLLOW-UP)

SYSTEM USABILITY SCALE

Each item below is scored by respondents on a scale from 1 = “Strongly agree” to 5 = “Strongly disagree” [57].

- I think that I would like to use this system frequently.
- I found the system unnecessarily complex.
- I thought the system was easy to use.
- I think that I would need the support of a technical person to be able to use this system.
- I found the various functions in this system were well integrated.
- I though there were too much inconsistency in this system.
- I would imagine that most people would learn to use this system very quickly.
- I found the system very cumbersome to use.
- I felt very confident using the system.
- I needed to learn a lot of things before I could get going with the system.

EXPERIENCE OF SUPPORT PROVIDED

- Overall, how well suited do you believe that the support was to your needs? (1 = “Not very well” to 5 = “Very well”). Please leave a comment describing your needs and how the intervention matched or did not match them (Free-text).
- Do you believe that the content in the dashboard would be helpful for people that want to reduce their consumption? (1 = “Not very helpful” to 5 = “Very helpful”)

- Do you believe that the content in the SMS messages would be helpful for people that want to reduce their consumption? (1 = “Not very helpful” to 5 = “Very helpful”)
- If you were to continue using the support, for how much longer would you want to use it?
  - I would use it for one to two more months
  - I would use it for three to six more months
  - I would use it for more than six months
  - I do not want to use it any more
  - I do not know
- Would you recommend this intervention to a friend who expresses a wish to reduce their alcohol consumption?
  - Yes
  - No
  - I do not know

CONTROL GROUP EXPERIENCE (FOUR-MONTH FOLLOW-UP)

Questions asked to the control group at the end of the trial (four-month follow-up) prior to giving them access to the intervention.

You were part of the group that was given access to information before being given access to the mobile phone based support. Out of the options listed below, which best describes your immediate reaction and your later actions. Please also leave a comment to explain your response.

- Immediate response:
  - Neither positive or negative. It did not really matter for me.
  - Interested to check out the information.
  - Frustration, irritation or disappointment. I was ready for extra support to reduce my consumption.
  - Pleased.
  - I do not know.
- Actions:
  - I decided to motivate myself and reduce my alcohol consumption.
  - I decided to continue my current consumption level, and to reduce my consumption once the initial phase was over.
  - I found other support that I used to reduce my consumption (please leave a comment on which support you used).
  - I gave up on the idea of reducing my alcohol consumption.
- Did you look at the information given and did you find it useful?
  - I looked at the information and found it useful to think about my drinking.
  - I looked at the information and found that it was not useful.

- I did not look at the information.
- I do not know.
